# Sources of vitamin D and determinants of serum 25-hydroxyvitamin D in Finnish adolescents

**DOI:** 10.1007/s00394-022-03039-y

**Published:** 2022-11-09

**Authors:** Sonja Soininen, Aino-Maija Eloranta, Ursula Schwab, Timo A. Lakka

**Affiliations:** 1 Institute of Biomedicine, School of Medicine, University of Eastern Finland, Kuopio, Finland; 2 Social and Health Center, Varkaus, Finland; 3 Institute of Public Health and Clinical Nutrition, School of Medicine, University of Eastern Finland, Kuopio, Finland; 4 Department of Medicine, Endocrinology and Clinical Nutrition, Kuopio University Hospital, Kuopio, Finland; 5 Department of Clinical Physiology and Nuclear Medicine, School of Medicine, Kuopio University Hospital, University of Eastern Finland, Kuopio, Finland; 6 Kuopio Research Institute of Exercise Medicine, Kuopio, Finland

**Keywords:** Vitamin D, 25-Hydroxyvitamin D, Adolescents, Diet, Supplements, Fortification

## Abstract

**Purpose:**

To study the intake and sources of vitamin D and determinants of serum 25-hydroxyvitamin D (S-25(OH)D) in Finnish adolescents.

**Methods:**

We studied 265 adolescents (117 girls) aged 15–17 years attending 8-year examinations of the PANIC Study, assessed diet using food records and other lifestyle factors by questionnaires, and analyzed S-25(OH)D by chemiluminescence immunoassay and determinants of S-25(OH)D using multivariate linear regression.

**Results:**

Mean (standard deviation) of total vitamin D intake from food and supplements was 19.2 (13.1) µg/d, and that of dietary vitamin D intake was 9.9 (5.4) µg/d. Milk fortified with vitamin D was the main dietary source of vitamin D, providing 45% of daily intake. Altogether, 29% of the adolescents used no vitamin D supplements and 25% did not meet the recommended total vitamin D intake of 10 µg/d. Mean (standard deviation) of S-25(OH)D was 62.0 (18.8) nmol/l, and S-25(OH)D was < 50 nmol/l in 29.5% of the adolescents. Vitamin D intake from supplements was the main determinant of S-25(OH)D (*β* = 0.465, *p* < 0.001), followed by consumption of milk products (*β* = 0.251, *p* < 0.001), consumption of meat products (*β* = 0.179, *p* = 0.002), travels to sunny countries (*β* = 0.178, *p* = 0.002), and average daylight time (β = 0.162, *p* = 0.004).

**Conclusion:**

Most of the adolescents had vitamin D intake at the recommended level, although a fourth did not meet the recommended total vitamin D intake of 10 µg/d and almost a third had S-25(OH)D < 50 nmol/l. More attention should be paid to the sufficient intake of vitamin D in adolescents who do not use vitamin D supplements or fortified milk products.

**Trial registration:**

ClinicalTrials.gov: NCT01803776, registered March 3, 2013.

## Introduction

Vitamin D is essential in childhood growth and bone mineralization [1]. Vitamin D may also have beneficial effects on other health outcomes than bone health, including respiratory infections, autoimmune diseases, cardiovascular risk factors, some types of cancer, and mortality, even though evidence on these health effects remains inconclusive [2, 3].

The recommendations for vitamin D intake and the definitions of vitamin D deficiency are based on a serum concentration of 25-hydroxyvitamin D (25(OH)D) [2]. The limit for vitamin D deficiency varies between 25 and 50 nmol/l, and the lower limit for sufficiency between 50 and 75 nmol/l [1, 4–9]. Such a variation in the recommendations for serum 25(OH)D concentration is partly related to different target groups. Moreover, some recommendations take only the effects of vitamin D on bone health into account, whereas others consider also other possible health effects. The recommendations for vitamin D intake targeted for general populations of different ages typically vary between 10 and 20 µg/d [6–9], and the recommendations aiming at higher serum levels above 75 nmol/l vary between 10 and 50 µg/d for different age groups [5]. The international comparison of serum 25(OH)D levels across studies has been challenging due to differences between serum 25(OH)D assays. The Vitamin D Standardization Program was therefore established in 2010 as an international collaborative effort to standardize the laboratory measurement of vitamin D status so that the results would be comparable and independent of the location and laboratory procedures used [10].

There are few natural sources of vitamin D, including fish, egg yolk, and some mushroom [7], and certain foods may therefore need to be fortified to increase vitamin D intake at the population level [7, 11]. Accordingly, the recommendation for vitamin D fortification of liquid milk products was increased from 0.5 to 1 µg/100 g and for spreads from 10 to 20 µg/100 g in Finland in 2010 [12]. Moreover, the Finnish recommendation for vitamin D supplement use was increased to 7.5 µg/d throughout the year for children and adolescents aged 2–17 years in 2011, and the recommendation for total vitamin D intake was increased to 10 µg/d for individuals aged 2–74 years in 2014 [13]. We found that more than half of the Finnish children 6–8 years of age had insufficient vitamin D intake and fifth of the children had a serum 25(OH)D concentration < 50 nmol/l at the baseline examinations of the Physical Activity and Nutrition in Children (PANIC) Study in 2007–2009 [14], which was prior to the increases in Finnish recommendations for vitamin D intake, supplementation use, and fortification. However, after these changes in the Finnish recommendations, the total intake of vitamin D and serum 25(OH)D concentrations have increased in adults [15–17], and total vitamin D intake has been sufficient in the majority of children [18, 19].

Adolescents appear to have a higher risk of low serum 25(OH)D compared with children and adults [20, 21], and it is important to understand why adolescents do not meet the vitamin D recommendations. However, there are no reports on vitamin D intake and serum 25(OH)D concentrations in adolescents after the increases of the recommended vitamin D intake, supplement use, and fortification in Finland. For these reasons, we studied total and dietary vitamin D intake, vitamin D supplementation use, the sources of vitamin D, serum 25(OH)D concentrations, and the cross-sectional determinants of serum 25(OH)D in a population-based sample of Finnish adolescents participating in the 8-year examinations of the PANIC Study in 2016–2018 using a standardized assay to measure serum 25(OH)D concentrations.

## Methods

### Study design and participants

The present analyses are based on the cross-sectional data from the 8-year examinations of the PANIC Study that is an 8-year controlled lifestyle intervention study aimed at investigating the effects of a combined diet and physical activity (PA) intervention on cardiometabolic risk factors in a population-based sample of children from the city of Kuopio, Finland. The details of the study protocol, including sample size calculations, participant recruitment, interventions, and assessments, have been described in detail previously [22, 23]. Briefly, we invited 736 children 6–9 years of age who started the first grade in 16 primary schools of Kuopio in 2007–2009 to the baseline examinations of the PANIC Study, and 504 children were included in the lifestyle intervention study. The participants did not differ in age, sex, or body mass index-standard deviation score (BMI-SDS) from all children who started first grade in the City of Kuopio in 2007–2009 based on data from the standard school health examinations performed for all Finnish children before the first grade. The lifestyle intervention was focused on improving diet quality, increasing PA, and decreasing sedentary time according to the Finnish dietary and PA recommendations [24, 25] and was not specifically targeted on increasing total vitamin D intake. Of the 504 children who participated in the baseline examinations, 277 (55%) attended the 8-year examinations between January 2016 and January 2018. Those who participated in the 8-year examinations did not differ in age, BMI-SDS, vitamin D intake from food or supplements, or the distribution of sex or study groups at baseline from those who dropped out (data not shown). Altogether 265 adolescents (117 girls, 148 boys) had data on serum 25(OH)D, had no diseases or medications known to affect serum 25(OH)D, and were thus included in these analyses based on the cross-sectional data from the 8-year examinations of the PANIC Study. Of these adolescents, 97.3% were Caucasian.

### Assessment of food consumption and nutrient intake

We assessed the consumption of foods, energy intake, and the dietary intake of vitamin D using food records of 4 days in 213 (96%) adolescents or 3 days in 8 (4%) adolescents [23]. The clinical nutritionists who were trained based on the protocol of the study gave the instructions about filling out the food records to the participants at the research site during the study visits. The adolescents were instructed to record their food and drink consumption using household or other measures, such as tablespoons, deciliters, and centimeters. They were also provided a picture booklet of portion sizes to help estimate portion sizes. The clinical nutritionists checked the returned food records, together with the adolescents and completed any missing information. If any missing information remained, the researchers used standard portion sizes and quality details according to the study protocol. Food consumption and nutrient intakes were assessed using the Micro Nutrica^®^ dietary analysis software, version 2.5 (The Social Insurance Institution of Finland, Turku, Finland). The software is based on national analyses and international food composition tables [26]. Moreover, a clinical nutritionist updated the software by adding new food products with their actual nutrient content based on the updated data in the Finnish food composition database [27] or received from the food producers.

Milk was generally fortified with vitamin D with mostly 1 μg/100 g as recommended by the National Nutrition Council [12], but some milk products contained even 2 μg of vitamin D/100 g. In Finland, vitamin D fortification of foods is voluntary, except for fat-free homogenized milk (including organic fat-free homogenized milk), for which fortification of at least 1 µg/d has been mandatory since 2016 [28]. Some of the yoghurts and other sour milk products were fortified with vitamin D mostly with 1 μg/100 g. Vegetable oil-based spreads were generally fortified with vitamin D with 20 μg/100 g according to the recommendations [12]. Other fat products included mainly vegetable oil-based baking products, many of which are fortified with vitamin D in Finland, and coconut oil that is not fortified with vitamin D. Fish products included fresh fish, shellfish and processed fish. Plant based milk-type products, such as oat and soya drink, are usually fortified with vitamin D. Vitamin supplements were not included in the food records.

### Assessment of supplement use

The use of vitamin and mineral supplements was assessed by a questionnaire in which we asked if the adolescents had used any supplement containing vitamin D during the past year, the name of the supplement product used, the dose of vitamin D in the supplement in µg, how many times per week that supplements was used, in which month did the adolescent begin to use the supplement and in which month did the adolescent quit to use the supplement (if not used year-round). Vitamin D supplements and multivitamin supplements containing vitamin D were combined for the analyses. The average daily dose of vitamin D during a year was calculated by combining the information on the frequency of supplement use, the estimated number of weeks per year of supplement use, and the dosage by one researcher (SS).

### Assessment of physical activity and sedentary time

PA and sedentary time were assessed by the PANIC Physical Activity and Hobby Questionnaire. The types of PA included commuting to and from school, physical education at school, PA during recess, organized sports, competitions and games in sports, organized exercise other than sports, and unsupervised PA. The adolescents were asked to report whether they had done specific types of PA during the previous year. They were then asked to report the number of months of each PA per year, the number of sessions of each PA per week, and the duration of a single session of each type of PA separately on weekdays and weekend days. The total amount of each PA was calculated and expressed in hours per day. The amount of total PA was calculated by summing the amount of each PA type and was expressed in hours per day.

Sedentary time included watching TV and videos, using the computer or a tablet, playing video games, using a mobile phone and playing mobile games, listening to music, playing musical instruments, singing, reading, writing, drawing, doing arts and crafts, playing board and card games, cooking and baking, and sitting and lying for a rest. The amount of sedentary time was calculated by summing the amount of each type of sedentary time, weighted by the numbers of weekdays and weekend days, and was expressed in hours per day.

### Assessment of body size and composition

Body height and weight were assessed the children having fasted for 12 h. Body height was assessed using a wall-mounted stadiometer and body weight using the InBody^®^ 720 bioelectrical impedance device (Biospace, Seoul, South Korea), with the weight assessment integrated into the system. We computed age- and sex-standardized BMI-SDS using Finnish references [29]. Body fat percentage was measured in the supine position, empty-bladdered and in light clothing by dual-energy X-ray absorptiometry (DXA) using the Lunar DXA device (Lunar Prodigy Advance; GE Medical Systems).

### Assessment of other determinants of serum 25-hydroxyvitamin D concentration

Daylight time from sunrise to sunset in Kuopio, Finland, at latitude 62.89°N, was calculated as the average during three months before the blood sampling. The daylight time was provided by the Almanac Office, University of Helsinki. The seasons of blood sampling were determined as winter (December–February), spring (March–May), summer (June–August) and autumn (September–November). Travels to sunny countries within three months before the blood sampling (no, yes), sunscreen use (no, yes), skin color type (four categories according to Fitzpatrick [30] from light to dark), sun preference behavior (avoiding sun, not avoiding sun), race (Caucasian, non-Caucasian), parental education, and household income were assessed using questionnaires completed by the adolescents or their parents. Skin types I–II and III–IV were combined. Parental education was defined as the highest completed or ongoing degree of the parents and was categorized in two classes as occupational education, occupational institute or university of applied sciences, and university. Household income was reported to accuracy of 10 000 € and was categorized as ≤ 60000 €/y or > 60000 €/y.

### Measurement of serum 25-hydroxyvitamin D concentration

Venous blood samples were taken after 12-h overnight fasting. Blood was immediately centrifuged and stored at a temperature of −75 °C until biochemical analyses. Serum 25(OH)D concentration was analyzed by a chemiluminescence immunoassay the LIAISON^®^ 25 OH vitamin D TOTAL Assay (DiaSorin, Stillwater, MN) using an automatic LIAISON^®^ XL immunoanalyzer (DiaSorin). The assay range is from 10 to 375 nmol/l. Intra-assay variation was 1.7% at the concentration range of 21–103 nmol/l. Inter-assay variation was from 6.1 to 7.0%. The 25(OH)D analyses were performed in Eastern Finland Laboratory Centre Joint Authority Enterprise, which participates in the vitamin D External Quality Assessment Scheme and met the performance targets of this quality control system in January 2021. The LIAISON^®^ 25 OH vitamin D TOTAL Assay has received the certificate of the vitamin D Standardization-Certification Program [31].

### Statistical analyses

Statistical analyses were performed using the IBM SPSS Statistics^®^, Version 27 (IBM Corp., Armonk, NY, USA). The normality of distributions of the variables was verified visually and by the Kolmogorov–Smirnov test. The *T* test for independent samples for continuous variables with normal distributions, the Mann–Whitney *U* test for continuous variables with skewed distributions and the Pearson χ2 test for categorical variables were used to examine differences in the basic characteristics between sexes. We calculated the contribution of each food group to the total intake of vitamin D using the population proportion method as defined by Krebs–Smith and coworkers [32]. Food groups that provided at least 4% of dietary intake of vitamin D were used in the linear regression analysis that was used to investigate the determinants of serum 25(OH)D concentration. Fish consumption was divided into two classes (yes, no) for regression models due to very skewed distribution. Body fat percentage was the strongest correlate of 25(OH)D among variables related to body composition and was used in regression analyses. The variables were first entered one by one into the models and then entered stepwise into the model to study whether they were independently associated with serum 25(OH)D concentration. Risk factors of having serum 25(OH)D concentration < 50 nmol/l were studied using logistic regression analysis adjusted for age and sex. For these analyses, vitamin D supplement use was divided into 0, 0.01–9.99 and ≥ 10 µg/d and other continuous variables were categorized into thirds. Differences and associations with a *p* value of < 0.05 were considered statistically significant.

## Results

### Characteristics of children

Boys had higher body weight and height, and lower body fat percentage, were physically more active and less likely to use sunscreen, had higher vitamin D intake from food and higher total vitamin D intake from food and supplements, higher serum 25(OH)D concentration, and a higher proportion of parents having university education than girls (Table 1). Boys consumed more milk products, fat products, meat products, grain products and other food but less fruits and berries than girls (Table 2).

**Table 1:** Characteristics of adolescents

	All^a^	Girls^a^	Boys^a^	*p* value
Age (years)	15.8 (0.4)	15.8 (0.4)	15.8 (0.5)	0.090
Parental education				
Occupational education, occupational institute or university of applied sciences	127 (51.6%)	68 (60.2%)	59 (44.4%)	0.013
University education	119 (48.4%)	45 (39.8%)	74 (55.6%)	
Household income				
< 60000 €/y	76 (32.9%)	38 (35.8%)	38 (30.4%)	0.380
≥ 60000 €/y	155 (67.1%)	68 (64.2%)	87 (69.6%)	
Body height (cm)	171.9 (8.5)	166.0 (5.6)	176.7 (7.4)	< 0.001
Body weight (kg)	62.6 (13.3)	58.2 (8.9)	66.0 (15.2)	< 0.001
BMI-SDS	−0.03 (1.00)	0.08 (0.85)	−0.12 (1.11)	0.097
Body fat percentage	23.3 (10.7)	30.2 (7.1)	17.5 (9.6)	< 0.001
Skin type				
I–II always burns, never tans or often burns, sometimes tans	86 (32.8%)	44 (38.3%)	42 (28.6%)	0.097
III–IV sometimes burns, often tans or never burns, always tans	176 (67.2%)	71 (61.7%)	105 (71.4%)	
Physical activity (h/d)	2.5 (2.0)	2.0 (1.1)	3.0 (2.4)	< 0.001
Sedentary time (h/d)	8.6 (4.0)	8.6 (3.8)	8.6 (4.1)	0.967
Average daylight time during 3 months before blood sampling (h/d)	12.2 (4.0)	12.3 (0.4)	12.1 (0.3)	0.708
Season of blood sampling				
Winter	43 (16.2%)	21 (17.9%)	22 (14.9%)	0.208
Spring	103 (38.9%)	39 (33.3%)	64 (43.2%)	
Summer	45 (17.0%)	18 (15.4%)	27 (18.2%)	
Autumn	74 (27.9%)	39 (33.3%)	35 (23.6%)	
Travels to sunny countries				
No	229 (86.7%)	99 (85.3%)	130 (87.8%)	0.553
Yes	35 (13.3%)	17 (14.7%)	18 (12.2%)	
Sunscreen use				
No	99 (37.0%)	30 (25.6%)	69 (46.6%)	< 0.001
Yes	166 (62.6%)	87 (74.4%)	79 (53.4%)	
Sun preference behavior				
Avoiding sun	22 (8.3%)	13 (11.1%)	9 (6.1%)	0.141
Not avoiding sun	243 (91.7%)	104 (88.9%)	139 (93.9%)	
Vitamin D intake from food (µg/d)	9.9 (5.4)	8.0 (4.7)	11.7 (5.5)	< 0.001
Vitamin D supplement use				
No	76 (28.7%)	35 (29.9%)	41 (27.7%)	0.693
Yes	189 (71.3%)	82 (70.1%)	107 (72.3%)	
Average vitamin D intake from supplements during a year (µg/d)	8.6 (9.6)	9.4 (9.3)	8.6 (13.6)	0.772
Total vitamin D intake from food and supplements	19.2 (13.1)	16.1 (11.1)	22.1 (14.1)	0.001
Serum 25(OH)D (nmol/l)	62.0 (18.8)	59.0 (15.9)	64.4 (20.5)	0.015

The values are means (standard deviations) for continuous variables with normal distributions, medians (interquartile ranges) for continuous variables with skewed distributions (only vitamin D intake from supplements), or numbers (percentages) of adolescents for categorical variables. Differences between girls and boys were tested with the independent samples *t* test for continuous variables with normal distributions, the Mann–Whitney *U* test for continuous variables with skewed distributions, and the Pearson’s χ^2^ test for categorical variables

BMI-SDS, body mass index standard deviation score calculated using Finnish reference values [29]; 25(OH)D, 25-hydroxyvitamin D

^a^*n* varies from 177 to 265:

*n* = 265, 117 girls and 148 boys: sex, age, height, weight, BMI-SDS, waist circumference, physical activity, average daylight time, season of blood sampling, sunscreen use, sun preference behavior, serum 25(OH)D

*n* = 264, 116 girls and 148 boys: travels to sunny countries

*n* = 262, 115 girls and 147 boys: skin type

*n* = 261, 116 girls and 145 boys: sedentary time

*n* = 254, 116 girls and 138 boys: body fat percentage

*n* = 246, 113 girls and 133 boys: parental education

*n* = 231, 106 girls and 125 boys: household income

*n* = 221, 108 girls and 113 boys: vitamin D intake from food

*n* = 212, 102 girls and 110 boys: total vitamin D intake from food and supplements

*n* = 177, 76 girls and 101 boys: average vitamin D intake from supplements during a year among supplement users

**Table 2:** Food consumption in adolescents

Food (g/d)	All *n* = 221	Girls *n* = 108	Boys *n* = 113	*p* value
*Milk products*				
Mean (SD)	578.1 (363.1)	471.7 (305.6)	679.8 (385.2)	< 0.001
Median (IQR)	501.0 (492.4)	396.9 (412.4)	606.0 (500.7)	
Milk				
Mean (SD)	434.7 (322.5)	331.0 (263.6)	533.9 (343.0)	< 0.001
Median (IQR)	383.3 (438.1)	293.1 (416.3)	488.8 (481.5)	
Skimmed milk (< 1% of fat)				
Mean (SD)	340.9 (322.0)	250.7 (251.8)	427.0 (357.4)	< 0.001
Median (IQR)	296.1 (454.1)	203.1 (434.4)	375.0 (534.6)	
Fat-containing milk (≥ 1% of fat)				
Mean (SD)	93.9 (164.8)	80.3 (140.2)	106.8 (185.0)	0.465
Median (IQR)	26.6 (94.16)	22.0 (84.9)	30.9 (102.1)	
Sour milk products				
Mean (SD)	76.0 (95.8)	68.5 (85.6)	83.1 (104.5)	0.664
Median (IQR)	50.0 (112.5)	43.9 (87.5)	50.0 (134.8)	
Other dairy products^a^				
Mean (SD)	67.4 (51.9)	72.2 (59.6)	62.8 (43.0)	0.181
Median (IQR)	54.4 (55.2)	56.0 (65.6)	52.5 (50.8)	
*Fat products*				
Mean (SD)	33.2 (16.6)	29.0 (14.2)	37.2 (17.7)	< 0.001
Median (IQR)	29.8 (22.0)	26.7 (16.6)	36.0 (24.3)	
Vegetable oil-based spreads				
Mean (SD)	11.7 (13.2)	9.6 (8.6)	13.7 (16.2)	0.781
Median (IQR)	8.0 (14.1)	7.5 (11.6)	8.5 (20.7)	
Butter and butter–oil mixtures				
Mean (SD)	8.4 (9.1)	7.1 (7.6)	9.6 (10.3)	0.094
Median (IQR)	5.3 (11.3)	3.9 (10.8)	6.3 (11.6)	
Vegetable oils^b^				
Mean (SD)	5.6 (5.3)	4.9 (4.0)	6.2 (6.2)	0.465
Median (IQR)	4.6 (6.0)	4.4 (5.3)	4.7 (7.6)	
Other fat products^c^				
Mean (SD)	7.5 (8.4)	7.3 (9.2)	7.6 (7.7)	0.419
Median (IQR)	5.0 (10.0)	5.0 (8.9)	4.9 (10.8)	
*Fish products*^d^				
Mean (SD)	19.9 (25.1)	17.1 (22.7)	22.5 (27.1)	0.140
Median (IQR)	10.0 (34.8)	4.3 (29.7)	15.4 (38.7)	
*Meat products*^e^				
Mean (SD)	131.7 (76.7)	95.0 (48.3)	166.7 (82.5)	< 0.001
Median (IQR)	117.3 (93.6)	93.6 (59.0)	153.6 (107.2)	
*Egg*				
Mean (SD)	18.0 (19.9)	16.7 (18.6)	19.3 (21.1)	0.320
Median (IQR)	12.0 (21.9)	10.4 (20.6)	13.5 (21.4)	
*Other food and drinks*^f^				
Mean (SD)	1355.7 (558.4)	1373.4 (539.3)	1338.8 (578.0)	0.646
Median (IQR)	1259.6 (695.0)	1253.7 (660.9)	1261.6 (706.0)	
Grain products				
Mean (SD)	218.7 (92.7)	202.0 (86.8)	234.7 (95.8)	0.009
Median (IQR)	205.3 (101.4)	189.8 (87.6)	225.1 (127.6)	
Vegetables				
Mean (SD)	208.9 (99.0)	205.4 (99.9)	212.3 (98.6)	0.576
Median (IQR)	191.9 (129.3)	189.6 (99.9)	193.7 (98.6)	
Fruits and berries				
Mean (SD)	108.2 (111.8)	135.6 (115.8)	81.9 (101.5)	< 0.001
Median (IQR)	74.0 (143.6)	121.0 (155.6)	42.2 (112.2)	
Drinks (other than milk products)				
Mean (SD)	732.9 (463.5)	753.2 (442.0)	713.5 (484.4)	0.526
Median (IQR)	28.4 (49.5)	711.5 (542.3)	617.9 (557.5)	
Sugar and sweets				
Mean (SD)	39.5 (37.4)	39.2 (31.1)	39.7 (42.8)	0.197
Median (IQR)	28.4 (49.5)	34.7 (44.9)	24.6 (53.5)	

The values are means (standard deviations) and medians (interquartile ranges). Differences between girls and boys were tested with independent samples *t *test for continuous variables with normal distributions (milk products, milk, other dairy products, fat products, meat products, other food and drinks, grain products, vegetables, drinks) and with Mann–Whitney *U* test for continuous variables with skewed variables (all other variables)

^a^Other dairy products included mainly cheese, creams, and ice cream

^b^Included liquid vegetable oil-based products

^c^Other fat products included mainly vegetable oil-based baking products

^d^Fish products included fresh fish, shellfish, and processed fish

^e^Meat products included red meat, sausage, and poultry

^f^Other food and drinks included grain products, vegetables, fruits, berries, other drinks than milk, and sugar and sweets

### Vitamin D intake from food and supplements

The mean total intake of vitamin D, including food and supplements, was 19.2 µg/d, and the mean intake of vitamin D from food was 9.9 µg/d (Table 1). Altogether, 71.3% of adolescents used supplements with vitamin D. The median of the average intake of vitamin D from supplements for those who used vitamin D supplements was 8.6 µg/d. The adolescents in the intervention group had higher mean (SD) dietary intake of vitamin D than those in the control group (10.5 [5.7] vs. 8.9 [4.8] µg/d, *p* = 0.025), but there was no difference in the median (IQR) of vitamin D intake from supplements among supplement users in the intervention group and the control group (8.7 [9.3] vs. 8.6 [11.1] µg/d, *p* = 0.707) or in the mean (SD) of total vitamin D intake (19.6 [12.3] vs. 18.6 [13.2] µg/d, *p* = 0.595). Of all adolescents, 75% (66.7% of girls, 82.7% of boys) met the Finnish recommendation of 10 µg/d for the total vitamin D intake from food and supplements [7, 13]. Total vitamin D intake was below the Finnish recommendation in 7.8% of the adolescents who used vitamin D supplements and in 69.5% of those who did not use supplements (*p* < 0.001). Altogether, 48.8% of the adolescents who used supplements met the target of daily vitamin intake of 10 µg/d already from food, whereas 30.5% of the adolescents who did not use supplements met this target (*p* = 0.016).

### Sources of vitamin D

Vitamin D supplements were the most important source of vitamin D, accounting for 48.4% of total vitamin D intake, followed by milk products (24.9%), fat products (16.8%), fish products (5.3%), meat products (2.6%), egg (1.2%) and other food (0.9%). Dietary sources of vitamin D (excluding supplements) are described in Table 3.

**Table 3:** Main dietary sources of vitamin D in adolescents^a^

		Dietary intake of vitamin D	
Food	Mean (SD), µg/d	Median (IQR), µg/d	Percentage^b^
*Milk products*	4.8 (3.6)	4.4 (5.0)	48.5
Milk	4.4 (3.5)	4.0 (4.7)	44.7
Skimmed milk (< 1% of fat)	3.6 (3.5)	3.0 (4.9)	35.9
Fat-containing milk (≥ 1% of fat)	0.9 (1.6)	0.2 (0.8)	8.8
Sour milk products	0.3 (0.6)	0.0 (0.4)	2.7
Other dairy products	0.1 (0.2)	0.1 (0.1)	1.1
*Fat products*	3.2 (2.5)	2.5 (2.6)	32.4
Vegetable oil-based spreads	2.2 (2.6)	1.4 (2.6)	22.0
Butter and butter–oil mixtures	0.8 (1.3)	0.0 (1.0)	7.5
Vegetable oils^c^	0.0 (0.1)	0.0 (0.0)	0.3
Other fat products	0.3 (0.4)	0.1 (0.4)	2.6
*Fish products*	1.0 (1.6)	0.2 (1.6)	10.1
*Meat products*	0.5 (0.5)	0.3 (0.5)	4.9
*Egg products*	0.2 (0.3)	0.1 (0.3)	2.3
*Other food and drinks*	0.2 (0.4)	0.1 (0.1)	1.7

*SD* standard deviation, *IQR* interquartile range

^a^The contribution to the intake of vitamin D from diet only (excluding supplements)

^b^The percentage contribution to the total intake of vitamin D calculated using population proportion method[32]

^c^Included liquid vegetable oil-based products

### Serum 25-hydroxyvitamin D concentration

**Fig. 1:**
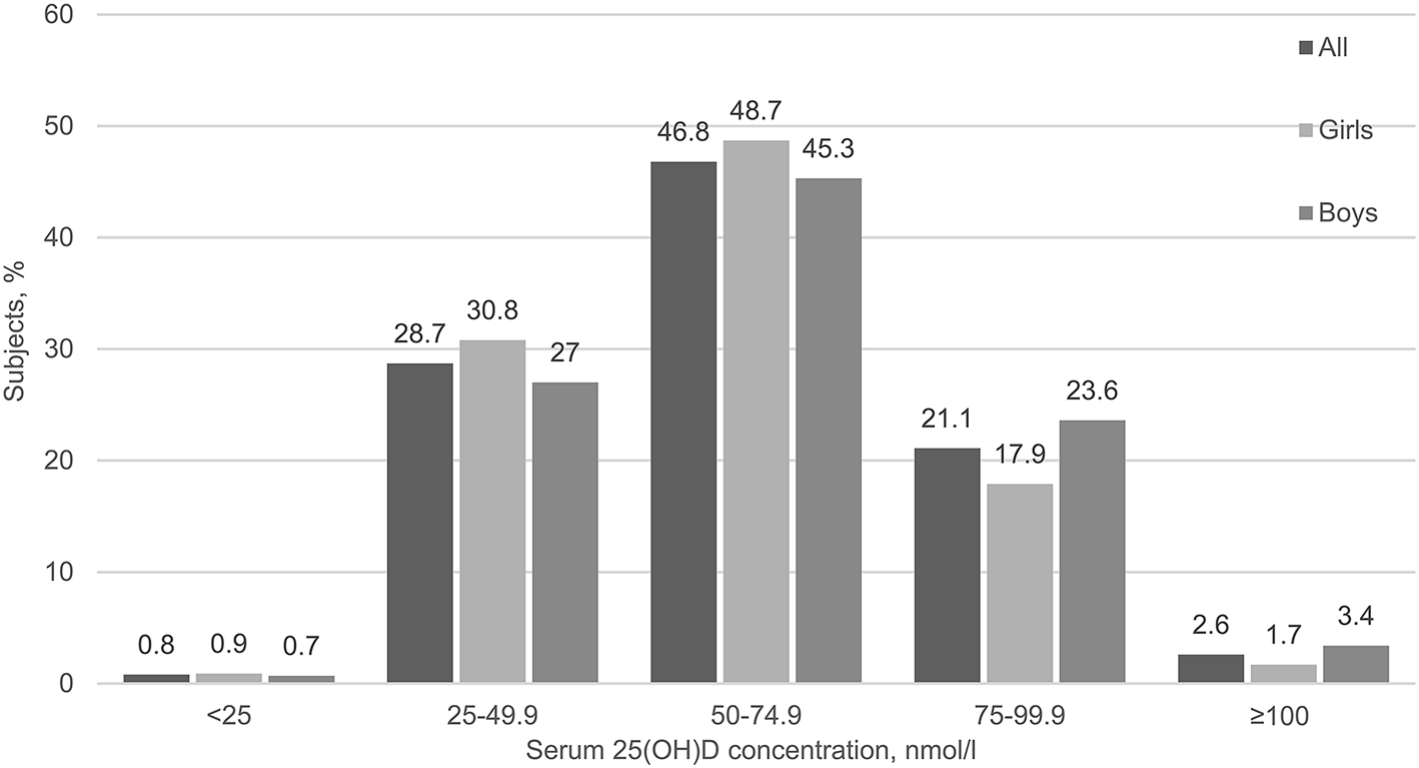
Distribution of serum 25-hydroxyvitamin D (25(OH)D) concentration

Serum 25(OH)D concentration varied between 16.4 and 153.0 nmol/l. The mean serum 25(OH)D was 62.0 nmol/l (Table 1). There was no difference in mean (SD) serum 25(OH)D concentration between adolescents in the intervention group and those in the control group (62.9 [18.9] vs. 60.6 [18.6], *p* = 0.325). Distribution of serum 25(OH)D concentration is illustrated in Fig. 1. Serum 25(OH)D was < 50 nmol/l in 29.5% of all adolescents, 31.6% of the girls and 27.7% of the boys and < 30 nmol/l in 1.9% of the adolescents, 1.7% of the girls and 2.0% of the boys. Altogether, 23.3% of the adolescents who used supplements had serum 25(OH)D < 50 nmol/l, whereas 44.7% of those who did not use them had serum 25(OH)D < 50 nmol/l (*p* = 0.001).

### Determinants of serum 25-hydroxyvitamin D concentration

In all adolescents, male sex, higher parental education, higher household income, lower body fat percentage, higher levels of PA, lower levels of sedentary time, longer average daylight time, travels to sunny countries, higher consumption of milk products, fat products, and meat products, and higher intake of vitamin D from supplements were associated with higher serum 25(OH)D without adjustments (Table 1, Model 1). Higher intake of vitamin D from supplements, higher consumption of milk products and meat products, travels to sunny countries, and higher daylight time were associated with higher serum 25(OH)D when all variables listed in Table 4 were entered simultaneously in the stepwise model (Table 4, Model 2).

**Table 4:** Determinants of serum 25-hydroxyvitamin D concentration in adolescents

	All^a^				Girls^a^				Boys^a^			
	Model 1		Model 2		Model 1		Model 2		Model 1		Model 2	
	*β*	*p* value	*β*	*p* value	*β*	*p* value	*β*	*p* value	*β*	*p* value	*β*	*p* value
Sex^b^	0.145	0.019										
Age (years)	0.114	0.065			0.132	0.155	0.225	0.016	0.084	0.313		
Parental education^c^	0.129	0.043			0.105	0.267			0.104	0.236		
Household income^d^	0.155	0.018			0.148	0.129			0.149	0.096		
Body fat percentage	−0.256	< 0.001			−0.193	0.038			−0.029	0.014		
Skin type^e^	−0.020	0.742			−0.007	0.945			−0.056	0.502		
Physical activity (h/d)	0.243	< 0.001			0.041	0.659			0.270	0.001		
Sedentary time (h/d)	−0.194	0.002			−0.158	0.089			−0.219	0.008		
Average daylight time (h/d)^f^	−0.158	0.010	0.162	0.004	0.091	0.332			0.209	0.011	0.153	0.046
Travels to sunny countries^g^	0.169	0.006	0.178	0.002	0.135	0.150	0.246	0.008	0.208	0.011	0.168	0.028
Sunscreen use^h^	0.117	0.058			0.048	0.608			0.210	0.010		
Milk products (g/d)	0.377	< 0.001	0.251	< 0.001	0.238	0.013	0.245	0.008	0.409	< 0.001	0.263	0.001
Fat products (g/d)	0.160	0.017			0.015	0.876			0.184	0.051		
Fish consumption (yes–no)	0.027	0.691			−0.122	0.208			0.123	0.196		
Meat products (g/d)	0.269	< 0.001	0.179	0.002	0.046	0.634			0.285	0.002	0.218	0.004
Vitamin D intake from supplements (µg/d)	0.434	< 0.001	0.465	< 0.001	0.316	0.001	0.406	< 0.001	0.491	< 0.001	0.504	< 0.001

The values are standardized regression coefficients (*β*) and *p* values from linear regression models by entering each variable separately in Model 1 and by entering all variables stepwise in Model 2

^a^*n* varies from 221 to 265 in different variables, resulting in 191 adolescents (92 girls, 99 boys) with all variables in Model 2:

*n* = 265, 117 girls and 148 boys: sex, age, height, weight, physical activity, average daylight time, season of blood sampling, sunscreen use

*n* = 264, 116 girls and 148 boys: travels to sunny countries

*n* = 262, 115 girls and 147 boys: skin type

*n* = 261, 116 girls and 145 boys: sedentary time

*n* = 254, 116 girls and 138 boys: body fat percentage

*n* = 253, 111 girls and 142 boys: vitamin D intake from supplements average during a year

*n* = 246, 113 girls and 133 boys: parental education

*n* = 231, 106 girls and 125 boys: household income

*n* = 221, 108 girls and 113 boys: dietary factors

^b^Sex; 1 = girl, 2 = boy

^c^Parental education; 1 = occupational education, occupational institute, or university of applied sciences, 2 = university education

^d^Household income; 1 =  < 60000 €/y, 2 =  ≥ 60000 €/y

^e^Skin types according to Fitzpatrick [30] combined in 2 classes: skin color type I or II = 1, III or IV = 2

^f^Average daylight time from sunrise to sunset during 3 months before blood sampling

^g^Travels to sunny countries within 3 months before blood sampling; 1 = no, 2 = yes

^h^Sunscreen use; 1 = no sunscreen use, 2 = sunscreen use occasionally or frequently

Among girls, lower body fat percentage, higher consumption of milk products, and higher intake of vitamin D from supplements were associated with higher serum 25(OH)D without adjustments (Table 1, Model 1). Higher vitamin D intake from supplements, higher consumption of milk products, travels to sunny countries, and an older age were associated with higher serum 25(OH)D in girls when all variables listed in Table 4 were entered simultaneously in the stepwise model (Table 4, Model 2).

Among boys, lower body fat percentage, higher levels of PA, lower levels of sedentary time, higher daylight time and travels to sunny countries, sunscreen use, higher consumption of milk products and meat products, and higher intake of vitamin D from supplements were associated with higher serum 25(OH)D without adjustments (Table 4, Model 1). Higher intake of vitamin D from supplements, higher consumption of milk products and meat products, travels to sunny countries and longer average daylight time were associated with higher serum 25(OH)D in boys when all variables listed in Table 4 were entered simultaneously in the stepwise model (Table 4, Model 2).

A higher dietary intake of vitamin D was associated with a higher serum 25(OH)D concentration in all adolescents (*β* = 0.289, *p* < 0.001), and in boys (*β* = 0.288, *p* = 0.002), but the association was not statistically significant in girls (*β* = 0.174, *p* = 0.072), without adjustments.

### Risk factors of having serum 25-hydroxyvitamin D concentration below 50 nmol/l

Adolescents who used vitamin D supplements at least 10 µg/d had 81% lower and those who used vitamin D supplements < 10 µg/d had 57% lower odds of having serum 25(OH)D concentration < 50 nmol/l than those who did not use supplements at all, adjusted for age and sex (Table 5). Adolescents who consumed milk at least 550 g/d (highest third) had 77% lower odds of having serum 25(OH)D < 50 nmol/l than those who consumed milk less than 250 g/d (lowest third). Adolescents who consumed meat at least 151 g/d (highest third) had 65% lower odds of having serum 25(OH)D < 50 nmol/l than those who consumed meat less than 92.4 g/d (lowest third). Adolescents with average daylight time of over 14.8 h/d during three months before blood sampling (highest third) had 72% lower and those with average daylight time of 10.0–14.8 h/d (middle third) had 63% lower odds of having serum 25(OH)D < 50 nmol/l than those with daylight time less than 10 h/d (lowest third). Adolescents whose blood samples were collected in summer had 79% lower, those whose blood samples were collected in spring had 58% lower, and those whose blood samples were collected in autumn had 55% lower odds of having serum 25(OH)D < 50 nmol/l than those whose blood samples were collected in winter. Adolescents who had travelled to sunny countries during three months before blood sampling had 76% lower odds of having serum 25(OH)D < 50 nmol/l than those without such travelling, and those who used sunscreen had 66% lower odds of having serum 25(OH)D < 50 nmol/l than those who did not use sunscreen. Adolescents whose parents had university education had 46% lower odds of having serum 25(OH)D < 50 nmol/l than those whose parents had lower education, and those whose parents reported household income ≥ 60000 €/y had 70% lower odds of having serum 25(OH)D < 50 nmol/l than those whose parents had lower income.

**Table 5:** Odds ratios (95% confidence intervals) of having serum 25-hydroxyvitamin D concentration below 50 nmol/l

	OR (95% CI)
Vitamin D supplement use, µg/d	
0.00	1.00 (reference)
0.01–9.99	0.43 (0.23–0.82)
≥ 10.00	0.19 (0.08–0.43)
* p* for linear trend	0.001
Milk consumption (g/d)	
< 250	1.00 (reference)
250–549	0.84 (0.43–1.67)
≥ 550	0.23 (0.10–0.55)
*p* for linear trend	0.001
Meat consumption (g/d)	
< 92.4	1.00 (reference)
92.4–151.0	0.66 (0.33–1.34)
≥ 151.0	0.35 (0.15–0.81)
* p* for linear trend	0.014
Average daylight time during 3 months before blood sampling (h/d)	
< 10.0	1.00 (reference)
10.0–14.8	0.37 (0.19–0.71)
> 14.8	0.28 (0.14–0.57)
* p* for linear trend	< 0.001
Season of blood sampling	
Winter (December, January, February)	1.00 (reference)
Spring (March, April, May)	0.42 (0.20–0.88)
Summer (June, August, July)	0.21 (0.75–0.60)
Autumn (September, October, November)	0.45 (0.21–0.99)
* p* for linear trend	0.081
Travels to sunny countries	
No	1.00 (reference)
Yes	0.34 (0.13–0.92)
* p* for linear trend	0.034
Sunscreen use	
No	1.00 (reference)
Occasionally or frequently	0.44 (0.25–0.78)
* p* for linear trend	0.005
Parental education	
Occupational education, occupational institute, or university of applied sciences	1.00 (reference)
University education	0.54 (0.30–0.96)
* p* for linear trend	0.035
Household income	
< 60,000 €/y	1.00 (reference)
≥ 60,000 €/y	0.30 (0.17–0.55)
* p* for linear trend	< 0.001

The values are from logistic regression models adjusted for age and sex

*CI* confidence interval, *OR* odds ratio

## Discussion

The results from our study in a population sample of Finnish adolescents showed that 25% did not meet the recommendation for total vitamin D intake from food and supplements of 10 µg/d [7, 13], 29% did not use vitamin D supplements, and 30% had serum 25(OH)D concentration < 50 nmol/l. Vitamin D intake from supplements and milk consumption were the most important determinants of vitamin D status, followed by consumption of meat products, travels to sunny countries, and average daylight time during three months before blood sampling. Milk and vegetable oil-based spreads, which are mostly fortified with vitamin D in Finland, were the main dietary sources of vitamin D. Using vitamin D supplements, consuming milk over 550 g/d, consuming meat and meat products over 151 g/d, having average daylight time over 10 h/d or travelling to sunny countries during three months before blood sampling, blood sampling in other seasons than winter, using sunscreen, having parents with higher education, and having higher household income were associated with lower odds of having serum 25(OH)D < 50 nmol/l.

The proportion of adolescents with serum 25(OH)D < 50 nmol/l in our Finnish study sample of 30% was lower than in other European study populations of school-age children and adolescents using standardized 25(OH)D concentrations [20], lower than in a large Swedish study population in adolescents [33] and almost same as in a Canadian study population of children and adolescents [21]. Only 0.8% of the adolescents in our study had serum 25(OH)D < 25 nmol/l and 1.9% had serum 25(OH)D < 30 nmol/l. However, the mean serum 25(OH)D was lower and the proportion of adolescents with serum 25(OH)D < 50 nmol/l was higher than among Finnish adults in 2011 [15] and in 2017 [17] and among 10-year-old Finnish children in 2013 [19] after the increase in the recommended vitamin D intake, supplementation, and fortification in Finland in 2010–2014 [7, 12, 13]. To the best of our knowledge, there are no previous data on serum 25(OH)D concentrations in Finnish adolescents after the updated national recommendations on vitamin D. The present data support the evidence that low vitamin D intake and vitamin D insufficiency remain common problems among children and adolescents in Nordic countries and elsewhere in Europe [3, 33, 34].

The average vitamin D intake among adolescents in the current study was 9.9 µg/d from food and 19.2 µg/d in total from food and supplements, which is well above the recommended 10 µg/d [7, 13], only slightly lower than in Finnish children 3–6 years of age [18], and higher than in school-age children and adolescents in other Nordic countries [34, 35]. Finnish children and adolescents 2–18 years of age are recommended to use vitamin D supplements 7.5 µg daily [13], and 71% of the adolescents in our study reported using supplements, even though many of them did not use supplements daily year-round. Vitamin D supplement use among adolescents was more common than in other Nordic countries [34]. However, 25% of all adolescents and up to 70% of those who did not use supplements did not meet the recommendation of total vitamin D intake 10 µg/d. In the most recent national FinDiet survey among Finnish adults in 2017, the mean vitamin D intake from food and supplements was higher than in adolescents of our study [17, 36].

The use of vitamin D supplements has been associated with higher serum 25(OH)D [14, 21, 37, 38] and a lower risk of having vitamin D deficiency in children and adolescents [39]. The use of vitamin D supplement has also been reported to reduce seasonal variation in serum 25(OH)D [40]. Vitamin D intake from supplements was the strongest determinant of serum 25(OH)D in the current study, and adolescents not using vitamin D supplements had higher odds of having low serum 25(OH)D than those using these supplements. The adolescents in the intervention group, the intervention having initiated eight years earlier in childhood, being based on the Finnish nutrition recommendations, and having resulted in a higher vitamin D intake than average in a Finnish diet [23, 36], had higher dietary intake of vitamin D than those in the control group, but there was no difference in serum 25(OH)D levels between the groups. Interestingly, a higher proportion of supplement users than non-users met the recommendation of total daily vitamin D intake 10 µg already from food without supplements, which suggests that those who eat healthier also follow the recommendations on supplement use better.

Fortified milk was the main dietary source of vitamin D, followed by fortified vegetable oil-based spreads, in adolescents participating in our study. They consumed more milk and less vegetable oil-based spreads and fish than adults in the FinDiet survey [36], which explains that distribution of dietary sources differed from that of adults [17]. Milk consumption was also the most important dietary determinant of serum 25(OH)D after the use of vitamin D supplements. Meat consumption was associated with serum 25(OH)D in boys but not in girls whereas consumption of fat products was not related to serum 25(OH)D in either sex even though fat products were a more important source of vitamin D. One explanation for this may be that meat consumption was high, boys consumed more meat than girls, and 25(OH)D that is found in meat has been suggested to have higher biopotency to increase serum 25(OH)D than D3 and especially D2, which are added in fortified fat products [7, 41]. Meat consumption was higher than recommended [13] especially in the boys. Because the production of meat and milk products has great environmental effects, alternative products including vitamin D are needed. However, the consumption of plant-based products fortified with vitamin D were too low to contribute to vitamin D intake in the current study. Up to 45% of the adolescents did not report consuming fish, and vitamin D intake from the natural sources of vitamin D such as fish and eggs [7] was low, which explains that they were not associated with serum 25(OH)D. The fish species eaten was reported in food records, but due to the low consumption of fish, we could not separate the consumption of fish species with higher vitamin D content in the linear regression analyses. Therefore, it is possible that the consumption of fish species containing most vitamin D [27] may have been too low to contribute 25(OH)D.

In Finland, milk and other dairy products are commonly used, which is one of the reasons why fortifying milk with vitamin D has been an effective strategy to increase vitamin D intake and serum 25(OH)D at the population level [15, 16]. Fortified milk products have been an important source of vitamin D and a major correlate of serum 25(OH)D also in other countries with national vitamin D fortification policy, such as in Canada and the USA, whereas their role has been smaller in countries where fortification is less systematic, such as in Sweden and Norway [42]. A recent national survey in Swedish adolescents found that adolescents with serum 25(OH)D < 30 nmol/l consumed less fortified products, even though the study was performed before implementing higher fortification policy in Sweden in 2018 [33]. We found that low serum 25(OH)D was more common and also dietary intake of vitamin D was lower in adolescents who did not use supplements than in those who used them. Current fortification policy seems effective in raising serum 25(OH)D among those who consume fortified products [15]. However, it is possible that a low-level fortification of several products may help increase vitamin D intake of the risk group that does not meet the recommendation of dietary vitamin D intake nor follow the recommendation to use supplements.

Girls had lower serum 25(OH)D than boys, which has been shown also in other studies among children and adolescents [14, 37, 43, 44]. Moreover, girls had lower vitamin D intake from food, which partly explains the sex difference. Another reason for the sex difference could be that girls had higher body fat percentage than boys. Obesity has been associated with decreased serum 25(OH)D in children and adolescents [21, 37, 44, 45], which may be due to storing vitamin D in adipose tissue [46], attenuated release of D3 from the skin into the circulation [47], impaired diet, or limited time spent outdoors. In the current study, higher body fat percentage was associated with lower serum 25(OH)D, but the association was explained by other factors. Boys had more PA, which may be associated with time spent outdoors that increases cutaneous synthesis of vitamin D and partly explain their higher serum 25(OH)D compared with girls. Higher levels of PA have been associated with higher serum 25(OH)D in children and adolescents [38, 44, 45]. Higher screen time or sedentary time has also been associated with a higher risk of vitamin D deficiency in children [37, 48] an in adolescents [33]. We found that higher levels of PA and lower levels of sedentary time were associated with higher serum 25(OH)D, but these associations were largely explained by other determinants.

Higher parental education and household income had weak positive associations with serum 25(OH)D in adolescents participating in our study. The adolescents whose parents had higher education or higher household income also had lower odds of having serum 25(OH)D < 50 nmol/l. In line with the findings of previous studies [21, 43, 48], the results of our study suggest that a higher socioeconomic position may be associated with a better vitamin D status.

Darker skin is known to produce less vitamin D than light skin [49], and adolescents with dark skin or a non-Caucasian race have been found to be at increased risk of low serum 25(OH)D [21, 44]. However, skin type was not associated with serum 25(OH)D in the current study in mostly Caucasian adolescents. Because the number of adolescents with very light and dark skin was very low, we combined skin types I–II and III–IV in the analyses, which may explain why an association between skin type and serum 25(OH)D was not found.

Sunlight has been stated to be the main determinant of vitamin D status at the global level, and several studies have shown seasonal variation in serum 25(OH)D with lowest concentrations observed in winter or early spring [14, 21, 40, 43, 44]. We found that longer average daylight time during three months before blood sampling was associated with higher serum 25(OH)D and that adolescents who had average daylight time < 10 h/d during this time period or were studied in winter had increased odds of having serum 25(OH)D < 50 nmol/l. Longer average daylight time was associated with higher serum 25(OH)D in boys, but not in girls. One reason for this may be a larger variation in exposure to sunlight and vitamin D production in the skin among boys part of whom spend a lot of time outdoors because of their physically active lifestyle. Travels to sunny countries were also associated with higher serum 25(OH)D. This association is also likely due to more time spent outdoors and higher cutaneous synthesis of vitamin D in lower latitudes than in Finland. Sunscreen use may reduce vitamin D production in the skin [49]. However, adolescents who used sunscreen had lower odds of having serum 25(OH)D < 50 nmol/l than those not using it, and such association of sunscreen in linear models was seen only in boys. One reason for this might be that boys who used sunscreen spent more time outdoors than girls using it. Another explanation for the sex difference could be that girls used sunscreen more frequently or in larger amounts than boys and that sunscreen use may have been sufficient to reduce the cutaneous synthesis of vitamin D induced by sunlight in girls but not in boys.

The strengths of this study include a population-based sample of adolescents whose vitamin D status and its determinants have been studied scarcely, the assessment of dietary vitamin D intake and other dietary factors using food records, the assessment of several other relevant determinants of serum 25(OH)D, and the measurement of serum 25(OH)D by an assay certified in Vitamin D Standardization-Certification Program [31]. High variation in daylight time across seasons due to high latitude allow us to study the association between daylight time and serum 25(OH)D, as sunlight is inadequate for vitamin D synthesis in the skin approximately half of the year. A weakness of this study is the assessment of vitamin D intake from supplements by a questionnaire, which may have underestimated or overestimated the use of vitamin D supplements. The number of adolescents who reported to avoid sun was also low, and we therefore were not able to analyze the association of sun preference behavior with serum 25(OH)D. We did not collect data on time spent outdoors that could only indirectly be estimated by the levels of PA that is partly performed outdoors. More health conscious and motivated adolescents may have participated in the study until the 8-year examinations compared with the drop-outs, which is a common phenomenon in studies with a long follow-up. However, the participants of this study did not differ in age, BMI-SDS, vitamin D intake from food or supplements, or the distribution of sex or study groups at baseline from those who dropped out. A large national survey is required to confirm if the results are generalizable nationwide as it is possible that there may be some local differences in intake, sources, and determinants of vitamin D. Finally, as cross-sectional data are used, causality of the associations cannot be proved.

## Conclusions

This study shows that 25–30% of the Finnish adolescents did not meet the recommendation for vitamin D intake from food and supplements, and 30% of the adolescents had serum 25(OH)D concentration < 50 nmol/l. Vitamin D supplement use and milk consumption were the main sources of vitamin D and the most important determinants of serum 25(OH)D. These findings emphasize that adolescents from Northern latitudes who do not use vitamin D supplements and consume fortified milk products are at increased risk of insufficient serum 25(OH)D concentrations.

## Data Availability

The PANIC Study is ongoing and therefore the data are not fully anonymized and, thus, not openly available. Part of the data can be shared by request.

## Abbreviations

25(OH)D: 25-Hydroxyvitamin D
BMI-SDS: Body mass index standard deviation score
DXA: Dual-energy X-ray absorptiometry
PA: Physical activity
PANIC Study: Physical Activity and Nutrition in Children Study
